# Effects of Hypoxia and Acidosis on Cardiac Electrophysiology and Hemodynamics. Is NHE-Inhibition by Cariporide Still Advantageous?

**DOI:** 10.3389/fphys.2020.00224

**Published:** 2020-03-19

**Authors:** Aida Salameh, Helena Zöbisch, Bianca Schröder, Jonas Vigelahn, Mandy Jahn, Getu Abraham, Johannes Seeger, Ingo Dähnert, Stefan Dhein

**Affiliations:** ^1^Heart Centre Clinic for Paediatric Cardiology, University of Leipzig, Leipzig, Germany; ^2^Faculty of Veterinary Medicine, Institute of Pharmacology, Pharmacy and Toxicology, University of Leipzig, Leipzig, Germany; ^3^Institute of Veterinary Anatomy, Histology and Embryology, University of Leipzig, Leipzig, Germany; ^4^Rudolf-Boehm-Institute for Pharmacology and Toxicology, University of Leipzig, Leipzig, Germany

**Keywords:** cariporide, hypoxia, acidosis, electrophysiology, langendorff

## Abstract

Hypoxia often leads to severe cardiac malfunctions. It is assumed that intracellular calcium overload is -inter alia- responsible for left ventricular (LV) deterioration. Inhibition of the sodium-proton exchanger (NHE), which finally inhibits/slows calcium overload, may ameliorate cardiac function. Our aim was to evaluate cariporide, an inhibitor of NHE1 in a Langendorff-perfused heart model. To discriminate a potentially different impact of extracellular acidosis and hypoxia we examined 48 Chinchilla Bastard rabbits divided into 8 experimental groups: control group (pH = 7.4, O_2_ = 100%) without or with cariporide (1 μM), acidosis group (pH = 7.0, O_2_ = 100%) without or with cariporide (1 μM), hypoxia group (pH = 7.4, O_2_ = 40%) without or with cariporide (1 μM) and hypoxia+acidosis group (pH = 7.0, O_2_ = 40%) without or with cariporide (1 μM). Hearts were subjected to acidotic/hypoxic conditions for 90 min followed by 60 min of reperfusion. Hypoxia and hypoxia+acidosis led to a severe deterioration of LV function with a decrease in LV pressure by about 70% and an increase of end-diastolic pressure from 6.7 ± 0.6 to 36.8 ± 5.4 (hypoxia) or from 7.0 ± 0.2 to 18.6 ± 4.1 (hypoxia+acidosis). Moreover, maximum contraction velocity decreased from about 1,800 mmHg/s to 600 mmHg/s during hypoxia ± acidosis and maximum relaxation velocity deteriorated from −1,500 mmHg/s to about −600 mmHg/s. During reperfusion hearts subjected to hypoxia+acidosis recovered faster than hearts subjected to hypoxia alone, reaching control levels after 5 min of reperfusion. Electrophysiologic analysis revealed an 1.2 fold increase in both dispersion of activation-recovery interval and in total activation time in the hypoxia ± acidosis group. Cariporide application significantly improved LV hemodynamics and electrophysiology in the hypoxia group but not in the group subjected to hypoxia+acidosis. Immunohistologic analysis of cardiac specimen revealed a significant increase of factors involved in hypoxia/reperfusion injury like nitrotyrosine and poly-ADP-ribose as well as apoptosis-inducing factors like AIF or cleaved-caspase 3 in LV after hypoxia ± acidosis. ATP was reduced by hypoxia but not by acidosis. Again, cariporide mitigated these processes only in the hypoxia alone group, but not in the group with additional acidosis. Acidosis without hypoxia only marginally disturbed LV function and electrophysiology, and was not affected by cariporide. Thus, our study demonstrated that several detrimental effects of hypoxia were mitigated or abrogated by acidosis and that NHE-inhibition improved only hypoxia-induced cardiac dysfunction.

## Introduction

Hypoxia and acidosis are relevant clinical pathophysiological mechanisms in situations like cardiac arrest. Both factors have been addressed in a recent published study of Spindelboeck et al. (2016), who analyzed the dynamic changes of blood oxygen, carbon dioxide and pH in resuscitated patients. The authors found out that low arterial partial oxygen pressure (pO_2_ < 75 mmHg) and high carbon dioxide pressure (CO_2_ > 69 mmHg) with acidotic pH (<7.05) were associated with a poor outcome. However, normal pO_2_ and slightly increased carbon dioxide values after restauration of circulation seemed to be beneficial for the perfusion of brain and heart (Eastwood et al., 2014; Helmerhorst et al., 2015). Thus, oxygen pressure and carbon dioxide seemed to be two independent variables influencing patient outcome after cardiac arrest. Nevertheless, increasing pCO_2_ is associated with low blood pH (respiratory acidosis), which was closely monitored and corrected in the study of Helmerhorst et al. (2015). On the other hand, the influence of low pO_2_ and low pH on the heart muscle has never been studied individually. Therefore, the first aim of our study was to evaluate the individual effects of hypoxia and acidosis and the combined effect of hypoxia+acidosis on the heart muscle.

As ischemic/hypoxic conditions result in intracellular acidosis, the NHE1 (besides the sodium-bicarbonate co-transporter) ensures the extrusion of excess protons, but simultaneously, to maintain electroneutrality, an equal amount of sodium is transported in the cell interior. This rise in intracellular sodium can activate the reverse mode of the sodium/calcium exchanger (NCX) which finally leads to a calcium and sodium overload (Ladilov et al., 1995; Salameh et al., 2002). The elevated calcium and sodium levels are responsible for induction of ventricular arrhythmias, for enhanced ventricular stiffness with elevation of left ventricular end-diastolic pressure and reduced left ventricular contractility. Moreover, -depending on the time of impaired oxygenation- cell apoptosis or necrosis will be induced, finally causing a pump failure of the heart (reviewed in Heusch and Gersh, 2017).

In contrast to hypoxia, acidosis seems to have a protective effect. In an animal infarct model Chi et al. (2019) demonstrated a better outcome of the hearts with smaller infarct sizes, higher ATP (adenosine tri-phosphate)-levels, and a better mitochondrial function. In isolated rat cardiomyocytes Ladilov et al. (1995) showed that prolonged acidosis can protect the cells from hypoxia-induced hypercontracture, which can be seen as a hint to a possible interaction between hypoxia and acidosis.

In the 1990ies, the amino-guanidine Hoe642 -designed by the Hoechst Company (Germany)- was introduced as a highly selective and potent inhibitor of the sodium/proton exchanger 1 (NHE1). It was proposed that Hoe642 (now named cariporide) should have beneficial effects on myocardial infarction, cardiac ischemia and reperfusion injury and during cardiosurgical interventions (Scholz et al., 1995). This has been shown in a model of cardioplegic arrest (Kevelaitis et al., 2005) and in animal models of ventricular fibrillation during resuscitation (Gazmuri et al., 2019). The cardioprotective effects of cariporide were also mimicked in mouse hearts by empagliflozin, a new antidiabetic drug of the group of the sodium-glucose cotransporter 2 inhibitors. This unexpected cardioprotective effect of empagliflozin was found to be based on an inhibition of NHE (Uthman et al., 2018a).

The NHE (the cardiac predominant isoform is the NHE1) is responsible for pH regulation, maintenance of cell volume and is also involved in disturbances of sodium and calcium homeostasis during pathophysiological situations like ischemia or hypoxia (Avkiran and Marber, 2002).

Experimental studies demonstrated, that inhibition of NHE1 delays intracellular pH recovery after ischemia (“acid freezing”) thereby reducing calcium overload and myocardial stunning, which will in turn improve ventricular performance (Strömer et al., 2000; Fujii et al., 2004).

A clinical study, the GUARDIAN trial, demonstrated that a significant benefit was not achieved by NHE-suppression but that certain patient groups may nevertheless profit from cariporide application (Théroux et al., 2000). This assessment led to another study -the EXPEDITION trial-, which was conducted on patients undergoing coronary artery bypass surgery. In this study, a risk reduction of death or myocardial infarction was achieved with cariporide. However, in the cariporide group mortality was increased within the first 30 days mainly due to cerebrovascular events (Mentzer et al., 2008). Despite these latter findings, the principle of NHE inhibition may be interesting with regard to the topics cardioprotection and prevention of reperfusion arrhythmias.

To analyze the role of NHE1-suppression during hypoxia and to dissect possible different effects of hypoxia and acidosis without hypoxia we investigated in a Langendorff-perfused rabbit heart model the hemodynamics and electrophysiology of this pathology without and with inhibition of NHE1 by cariporide.

In addition, we examined typical histochemical/biochemical effects of hypoxia such as nuclear AIF (apoptosis inducing factor)-translocation, PAR (poly-ADP ribose) formation, C3-cleavage (cleaved caspase 3), ATP content, and formation of nitrotyrosine (NT) in left and right ventricles.

## Materials and Methods

All experiments were performed in accordance with the ethical rules of the Council for International Organization of Medical Science and the German/European laws for animal welfare. The study was approved by our institutional ethical committee for animal welfare and the regional council of Leipzig named “Landesdirektion Sachsen” (reference number T03/17). The investigation conforms to the Directive 2010/63/EU of the European Parliament as well as to the Guide for the Care and Use of Laboratory Animals published by the US National Institutes of Health (NIH Publication No. 85–23, revised 1996).

We evaluated 48 Chinchilla Bastard rabbits (weight: 1,500–2,000g). Eight different experimental groups of n = 6 each, were defined: control group (pH = 7.4, O_2_ = 100%) without or with cariporide (1 μmol/L), acidosis group (pH = 7.0, O_2_ = 100%) without or with cariporide (1 μmol/L), hypoxia group (pH = 7.4, O_2_ = 40%) without or with cariporide (1 μmol/L) and hypoxia+acidosis group (pH = 7.0, O_2_ = 40%) without or with cariporide (1 μmol/L).

Langendorff-heart perfusion was done as previously published (Salameh et al., 2017). In brief, rabbits were exsanguinated in deep narcosis. The hearts were quickly prepared and connected to the Langendorff apparatus via the cannulated aorta. The pulmonary artery was also cannulated to measure coronary flow and a pressure balloon was inserted into the left ventricle for measurement of LVP (left ventricular pressure), EDP (end-diastolic pressure), dp/dtmax (maximum contraction velocity), and dp/dtmin (maximum relaxation velocity). Moreover, 254 electrodes were placed around the heart to derive epicardial ECGs (electrocardiograms) from right and left ventricular free wall as well as from the anterior and posterior wall (i.e., 64 single ECGs from each wall) according to Dhein et al. (1993). The hearts were perfused with Tyrode's solution (containing in mmol/L: Na^+^ 161, K^+^ 5.36; Ca^2+^ 1.8, Mg^2+^ 1.05; Cl^−^ 148, ${\text{HCO}}_{3}^{-}$ 23.8, ${\text{PO}}_{4}^{3-}$ 0.42, and glucose 11.1) and gassed with CO_2_ and O_2_ to achieve a pH of 7.4 and 100% oxygen (=normal Tyrode's solution) using a gas mixer (Stöckert, Munich, Germany). The Stöckert gas mixing device allows differential management of pH (via adjustment of CO_2_ flow) and oxygen (via adjustment of O_2_ flow and air). Both parameters (pH and O_2_) were measured several times thorough the experiment to ensure the correct experimental conditions.

After connection to the Langendorff apparatus, the hearts were allowed to beat spontaneously for 45 min. Thereafter, baseline hemodynamic parameters and ECGs were recorded and for the experiments with cariporide, the drug was then added to the Tyrode's solution. After another 15 min induction of acidosis, hypoxia or acidosis+hypoxia was started for in total 90 min. At time point 30, 60, and 90 min hemodynamic and electrophysiological parameters (TAT (total activation time), ARI (activation recovery interval), dispersion of ARI, PTP (peak-to-peak-amplitude), and VEC (vector field similarity of activation) were recorded.

For evaluation of the mapping data, the activation time points at each electrode were determined as t(dU/dt min) (Dhein et al., 1993). Next, the repolarization time points were determined as t(dU/dt max) during the T wave as described (Dhein et al., 1993). After automatic determination, activation and repolarization times were verified (or corrected if necessary) manually by the experimentator in a blinded fashion. From these data for each electrode, an activation recovery interval (ARI) was calculated as the difference between the time of activation and the time of repolarization, indicating the epicardial action potential duration. From the activation time points an activation sequence and the spread of epicardial excitation could be analyzed: for each electrode an activation vector was calculated from the activation times and the locations of the surrounding electrodes which were activated after the central electrode (i.e., a maximum number of 8), as described by Müller et al. (1991). These vectors give direction and apparent velocity of local activation. To compare the activation patterns of various heart beats the percentage of similar vectors (VEC) between heart beats in the various experimental phases compared with those under control conditions was determined (vectors deviating not more than 5° from their original direction were considered to be similar). The critical value beneath which arrhythmia often occurs (see above) for VEC similarity is 10% as determined in previous studies (Dhein et al., 1990, 1993).

Thus, the parameter VEC characterizes the geometry of the epicardial activation process and represents the beat similarity of the cardiac impulse as compared to heart beats under control conditions. Decreasing values for VEC indicate progressive deviation from the initial (control) activation pattern.

After 90 min of acidosis, hypoxia or hypoxia+acidosis without or with cariporide, reperfusion with normal Tyrode's solution (without or with cariporide) was initiated and hemodynamic and ECG measurements were performed after 5, 10, 20, 30, and 60 min of reperfusion. The control hearts were perfused with normal Tyrode's solution and evaluated at the same time points.

### Histology

For histological analysis, hearts were processed according to Salameh et al. (2015). Briefly, formalin-fixed hearts were embedded in paraffin and sectioned into 2 μm slices. Thereafter, heart slices were de-waxed, re-hydrated and specific primary antibodies to AIF (1:50, Santa Cruz, Heidelberg, Germany), cC3 (1:200, Cell Signaling, Frankfurt, Germany), PAR (1:600, Bio-Rad, Munich), and NT (1:50, Merck-Millipore, Darmstadt, Germany) were applied over night at 4°C. After several washing steps, secondary antibodies labeled with HRP (horseradish peroxidase) were administered for 1 h at room temperature. Thereafter, detection of signals was completed by application of the red chromogen AEC (3-amino-9-ethylcarbazole, Dako, Hamburg, Germany) according to the manufacturer's instruction. Nuclei of cardiomyocytes were counter-stained with haemalum. Specimen were embedded in glycerol-gelatin and investigated at 400x magnification with a Zeiss Axiolab microscope (Zeiss, Jena, Germany). Evaluation of cardiomyocytes was carried out by a blinded observer. We investigated right and left ventricular specimen separately and at least 50 cells per region were counted and the ratio of positive cells was evaluated in relation to the total number of counted cells. In that manner, 100 cells per heart and 600 cells per experimental group were counted.

### ATP Analysis by HPLC

Left ventricular specimen were homogenized on ice with 0.4 mmol/L perchloric acid and precipitated with KOH. The samples were centrifuged and supernatants were injected twice onto a pre-equilibrated RP18 column (LiChroCART, Merck, Darmstadt, Germany) as previously published (Salameh et al., 2015). For ATP detection a HPLC-apparatus from Knauer (Berlin, Germany) and an UV-detector (PDA Detector 2800, Knauer, Berlin, Germany) were used. Peaks were measured at 259 nm. Standard curves were generated with 4 concentrations of ATP (25-12.5-6.25-3.125μg/ml) and measured together with the ventricular samples.

## Statistical Analysis

The results are presented as mean ± standard error of means of n = 6 experiments. Hemodynamic and electrophysiological data were analyzed using ANOVA followed by *post-hoc* Tukey HSD test with Bonferroni correction for multiple measurements to detect statistical significance at a level of *p* < 0.05. For the statistical analyses Systat for Windows, version 13 (Systat Inc., Evanston, IL, USA) was used.

The non-parametric Kruskal–Wallis test was used to analyse the ATP data.

## Results

### Hemodynamic Parameter

**Figure 1:**
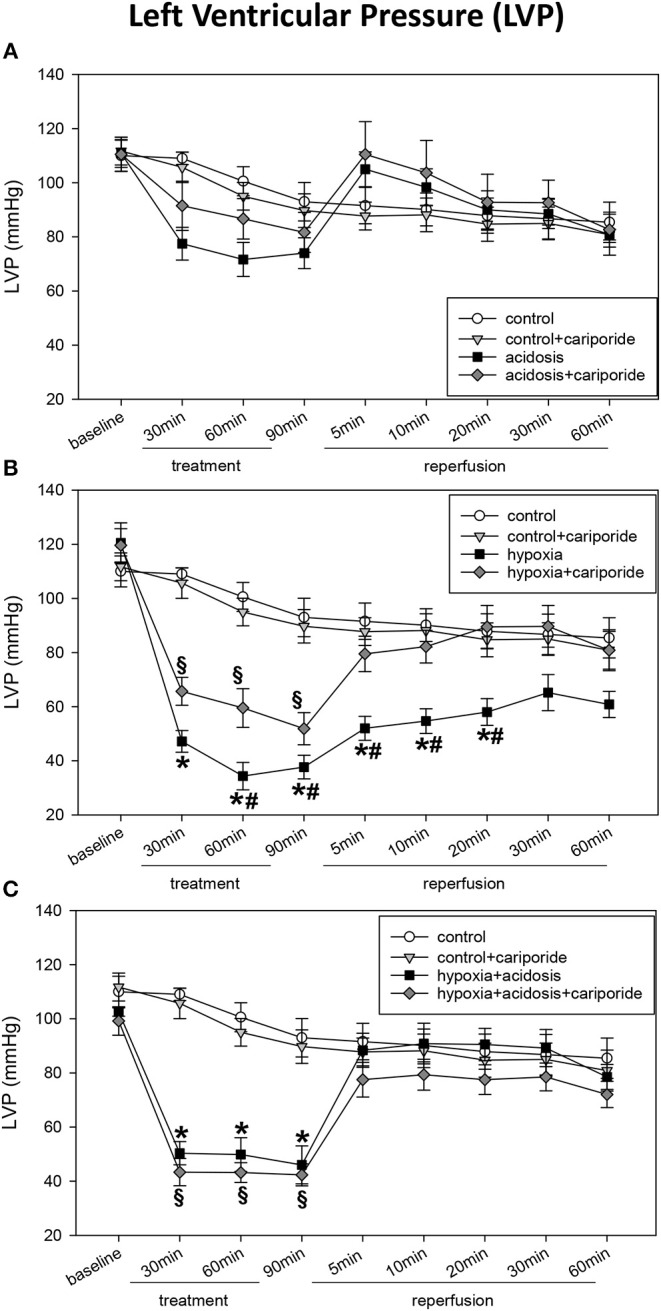
**(A)** Left ventricular pressure (LVP) in control hearts without or with cariporide 1 μmol/L and in hearts subjected to acidosis (pH = 7.0, O_2_ = 100%) without or with cariporide 1 μmol/L. **(B)** Left ventricular pressure (LVP) in control hearts without or with cariporide 1 μmol/L and in hearts subjected to hypoxia (pH = 7.4, O_2_ = 40%) without or with cariporide 1 μmol/L. **(C)** Left ventricular pressure (LVP) in control hearts without or with cariporide 1 μmol/L and in hearts subjected to hypoxia+acidosis (pH = 7.0, O_2_ = 40%) without or with cariporide 1 μmol/L. All data are given as means ± SEM. Significant differences (*p* < 0.05) vs. control are indicated by a *significant differences vs. control+cariporide by a ^**§**^ and significant differences vs. hypoxia + cariporide by a ^**#**^.

**Figure 2:**
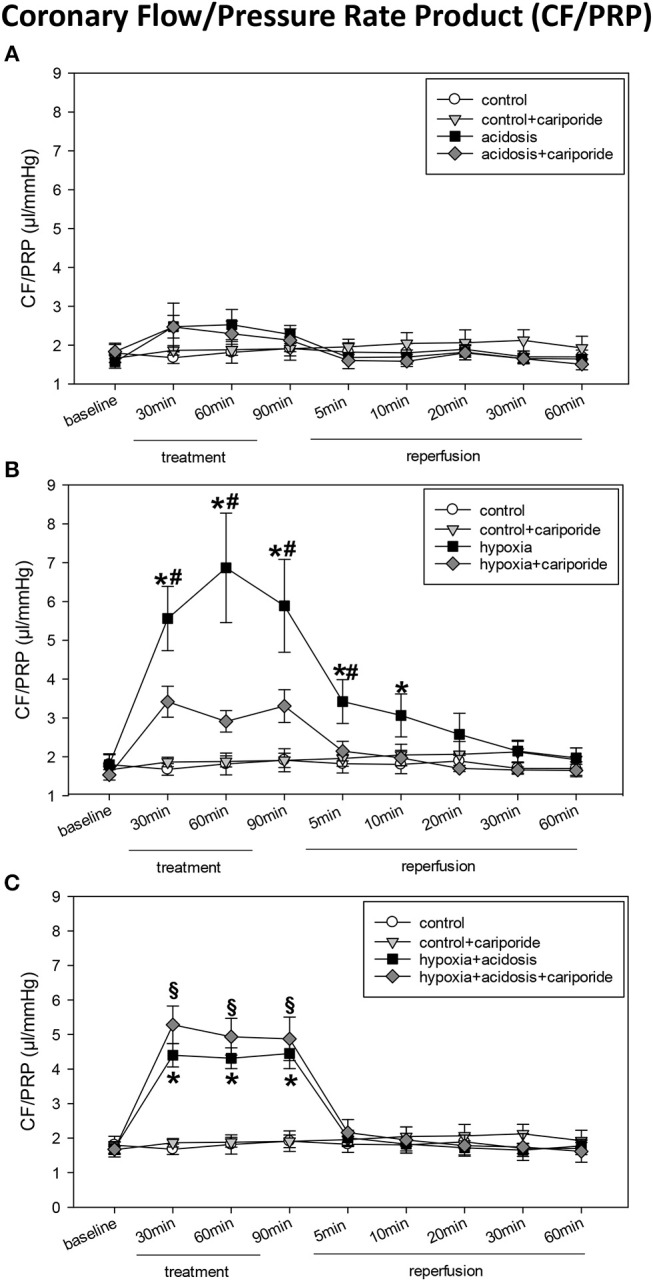
**(A)** Coronary flow/pressure rate product (CF/PRP) in control hearts without or with cariporide 1 μmol/L and in hearts subjected to acidosis (pH = 7.0, O_2_ = 100%) without or with cariporide 1 μmol/L. **(B)** Coronary flow/pressure rate product (CF/PRP) in control hearts without or with cariporide 1 μmol/L and in hearts subjected to hypoxia (pH = 7.4, O_2_ = 40%) without or with cariporide 1 μmol/L. **(C)** Coronary flow/pressure rate product (CF/PRP) in control hearts without or with cariporide 1 μmol/L and in hearts subjected to hypoxia+acidosis (pH = 7.0, O_2_ = 40%) without or with cariporide 1 μmol/L. All data are given as means ± SEM. Significant differences (*p* < 0.05) vs. control are indicated by a *significant differences vs. control+cariporide by a ^**§**^and significant differences vs. hypoxia+cariporide by a ^**#**^.

During acidosis (pH = 7.0) systolic LVP decreased from 111 ± 5.2 mmHg to 74 ± 5.7 mmHg. Dp/dtmax and PRP were significantly reduced after 90 min of acidosis: from 1,750 ± 92 to 1,067 ± 46 mmHg/s and from 17,695 ± 1,256 mmHg/min to 11,933 ± 874 mmHg/min, respectively. In contrast, CF/PRP slightly but not significantly increased during 90 min of acidosis (Figures 1, 2). LVP, PRP and dp/dtmax returned to baseline levels after 5 min of reperfusion with normal Tyrode's solution (pH = 7.4 and O_2_ = 100%). Application of cariporide improved ventricular function during acidosis. However, this improvement was not significant (Figure 1 and Table 1).

**Table 1:** Haemodynamik data.

	**Baseline**	**Treatment**			**Reperfusion**				
		**30 min**	**60 min**	**90 min**	**5 min**	**10 min**	**20 min**	**30 min**	**60 min**
Pressure rate product (PRP) in mmHg^*^min^**−1**^									
Control	19,279 ± 1,995	19,110 ± 1,032	18,050 ± 1,676	16,272 ± 1,956	15,789 ± 1,896	16,041 ± 1,825	15,456 ± 1,996	15,863 ± 1,892	16,119 ± 1,656
Control + cariporide	18,382 ± 916	15,885 ± 1,081	14,623 ± 1,020	13,531 ± 1,249	13,352 ± 829	13,205 ± 1,042	12,834 ± 1,161	11,814 ± 523	12,354 ± 1,283
Acidosis	17,695 ± 1,256	12,721 ± 1,004^*^	11,564 ± 1,055^*^	11,933 ± 874	16,801 ± 917	155,823 ± 1,099	14,244 ± 1,240	13,923 ± 931	12,553 ± 592
Acidosis + cariporide	16,514 ± 2,138	14,396 ± 2,463	13,583 ± 2,390	12,648 ± 2,185	16,575 ± 1,861	16,174 ± 2,446	14,070 ± 2,305	13,993 ± 1,602	14,117 ± 2,044
Hypoxia	19,276 ± 2,636	5,752 ± 832^*^	4,207 ± 908^*^^#^	4,597 ± 795^*^^#^	7,594 ± 1,655^*^^#^	7,896 ± 968^*^^#^	9,618 ± 1,201^*^^#^	10,598 ± 1,125^#^	11,633 ± 1,723
Hypoxia + cariporide	19,822 ± 1,424	9,747 ± 11,770^§^	10,520 ± 1,652	9,405 ± 1,674	14,455 ± 2,122	14,941 ± 1,868	16,213 ± 1,815	16,191 ± 1,817	16,042 ± 1,777
Hypoxia + acidosis	16,512 ± 1,011	7,182 ± 631^*^	7,385 ± 815^*^	7,042 ± 859^*^	15,677 ± 728	15,831 ± 907	16,286 ± 669	16,122 ± 585	14,007 ± 554
Hypoxia + acidosis + cariporide	16,376 ± 734	5,957 ± 1,101^§^	6,274 ± 1,251^§^	6,046 ± 1,187^§^	13,454 ± 1,217	14,334 ± 1,171	14,112 ± 1,241	15,120 ± 1,400	13,801 ± 1,130
**Maximum contraction velocity (dp/dt max) in mmHg/s**									
Control	1,642 ± 154	1,837 ± 76	1,736 ± 111	1,635 ± 130	1,585 ± 138	1,568 ± 137	1,551 ± 140	1,526 ± 147	1,551 ± 151
Control + cariporide	1,905 ± 57	1,686 ± 114	1,644 ± 113	1,521 ± 131	1,492 ± 122	1,500 ± 136	1,433 ± 147	1,433 ± 127	1,306 ± 137
Acidosis	1,750 ± 92	1,101 ± 66^*^	1,082 ± 66^*^	1,067 ± 46^*^	1,464 ± 45	1,437 ± 87	1,340 ± 95	1,269 ± 71	1,269 ± 73
Acidosis + cariporide	1,809 ± 113	1,475 ± 187	1,387 ± 214	1,396 ± 190	1,613 ± 159	1,573 ± 157	1,524 ± 137	1,446 ± 160	1,377 ± 159
Hypoxia	1,888 ± 103	816 ± 62^*^^#^	599 ± 54^*^^#^	620 ± 64^*^^#^	875 ± 75^*^^#^	929 ± 56^*^^#^	1,062 ± 93^*^^#^	1,200 ± 124	1,121 ± 81
Hypoxia + cariporide	1,873 ± 96	1,239 ± 126^§^	1,062 ± 112^§^	1,003 ± 128^§^	1,406 ± 151	1,436 ± 139	1,631 ± 150	1,623 ± 143	1,485 ± 121
Hypoxia + acidosis	1,505 ± 48	718 ± 51^*^	688 ± 54^*^	629 ± 68^*^	1,298 ± 63	1,298 ± 63	1,298 ± 48	1,289 ± 63	1,219 ± 68
Hypoxia + acidosis + cariporide	1,495 ± 45	551 ± 91^§^	561 ± 101^§^	561 ± 87^§^	1,160 ± 47	1,190 ± 51	1,200 ± 54	1,259 ± 58	1,210 ± 80
**Maximum relaxation velocity (dp/dt min) in mmHg/s**									
Control	−1,517 ± 80	−1,597 ± 52	−1,593 ± 101	−1,483 ± 99	−1,399 ± 114	−1,399 ± 113	−1,424 ± 120	−1,399 ± 116	−1,370 ± 127
Control + cariporide	−1,559 ± 38	−1,450 ± 59	−1,408 ± 66	−1,370 ± 78	−1,382 ± 75	−1,357 ± 90	−1,315 ± 95	−1,340 ± 89	−1,252 ± 107
Acidosis	−1,483 ± 80	−1,176 ± 92^*^	−1,146 ± 77^*^	−1,150 ± 69	−1,408 ± 47	−1,316 ± 67	−1,332 ± 77	−1,315 ± 78	−1,247 ± 15
Acidosis + cariporide	−1,524 ± 78	−1,337 ± 152	−1,249 ± 159	−1,229 ± 157	−1,308 ± 112	−1,405 ± 117	−1,292 ± 103	−1,306 ± 114	−1,236 ± 103
Hypoxia	−1,544 ± 88	−728 ± 71^*^	−541 ± 78^*^	−556 ± 79^*^	−777 ± 83^*^^#^	−890 ± 60^*^^#^	−959 ± 72^*^^#^	−1,082 ± 91	−988 ± 62^*^^#^
Hypoxia + cariporide	−1,586 ± 30	−974 ± 96^§^	−890 ± 104^§^	−806 ± 115^§^	−1,239 ± 142	−1,308 ± 109	−1,416 ± 120	−1,426 ± 103	−1,356 ± 89
Hypoxia + acidosis	−1,387 ± 20	−698 ± 44^*^	−688 ± 64^*^	−634 ± 83^*^	−1,239 ± 57	−1,239 ± 55	−1,268 ± 50	−1,278 ± 54	−1,180 ± 50
Hypoxia + acidosis+ Cariporide	−1,318 ± 62	−580 ± 77^§^	−580 ± 69^§^	−590 ± 68^§^	−1,052 ± 67	−1,092 ± 59	−1,121 ± 65	−1,111 ± 58	−1,052 ± 58
**End-diastolic pressure (EDP) in mmHg**									
Control	6.8 ± 0.64	8.1 ± 1.16	9.1 ± 0.96	9.3 ± 1.08	9.5 ± 0.90	8.8 ± 1.13	10.2 ± 1.37	10.7 ± 1.67	9.7 ± 1.10
Control + cariporide	7.9 ± 0.45	9.6 ± 0.69	9.2 ± 0.84	9.4 ± 0.78	9.6 ± 0.79	10.0 ± 1.09	10.0 ± 1.39	10.8 ± 1.73	10.8 ± 1.73
Acidosis	7.1 ± 0.67	9.1 ± 0.92	10.3 ± 1.74	11.0 ± 1.55	7.0 ± 1.05	7.4 ± 1.21	10.0 ± 1.10	9.5 ± 1.18	9.7 ± 1.40
Acidosis + cariporide	7.3 ± 0.72	8.1 ± 1.25	11.1 ± 0.98	10.8 ± 1.05	7.8 ± 1.80	7.9 ± 1.70	7.9 ± 1.98	8.4 ± 1.83	10.4 ± 2.55
Hypoxia	6.7 ± 0.58	28.8 ± 3.50^*^^#^	33.8 ± 3.67^*^^#^	36.8 ± 5.44^*^^#^	25.2 ± 3.23^*^^#^	241 ± 249^*^^#^	200 ± 306^*^^#^	18.7 ± 2.63^*^	18.4 ± 2.55^*^^#^
Hypoxia + cariporide	7.5 ± 0.36	17.4 ± 2.92	22.2 ± 1.80^§^	24.9 ± 1.77^§^	15.5 ± 2.77	14.9 ± 2.51	13.0 ± 2.24	13.1 ± 2.22	11.1 ± 2.61
Hypoxia + acidosis	7.0 ± 0.2	17.6 ± 3.05	18.5 ± 3.60^*^	18.6 ± 4.06	10.0 ± 0.74	9.2 ± 0.74	9.2 ± .074	9.6 ± 0.62	9.0 ± 0.74
Hypoxia + acidosis + cariporide	6.2 ± 0.48	19.5 ± 3.78^§^	16.8 ± 3.13	17.1 ± 2.38	8.0 ± 1.40	8.1 ± 0.98	8.2 ± 1.33	8.7 ± 1.78	7.2 ± 0.86

*All data are given as means ± SEM*.

* *significant differences vs. control*.

# *significant differences vs. hypoxia+cariporide*.

§ *significant differences vs. control+cariporide*.

Hypoxia (40% O_2_) und hypoxia+acidosis (40% O_2_+pH = 7.0) severely impaired left ventricular hemodynamics significantly. In case of hypoxia alone (with normal pH in the perfusion fluid) LVP was reduced by about 70% after 90 min. After reperfusion with normal Tyrode's solution LVP increased and after 20 min LVP values were not significantly different from control levels. The same was found analyzing the other ventricular contraction parameters: PRP and dp/dtmax decreased by about 75% (PRP) and by about 70% (dp/dtmax), respectively. Control levels were reached after 30 min reperfusion with normal Tyrode's solution (Figure 1 and Table 1). In accordance, CF/PRP increased significantly from 1.8 ± 0.28 μl/mmHg to 5.9 ± 1.2 μl/mmHg, and successively declined to control values after reperfusion (Figure 2).

Application of hypoxia+acidosis also led to a reduction in LVP, PRP, and dp/dtmaxin the same order of magnitude as hypoxia alone. However, after reperfusion baseline levels were already achieved within 5 min (Figure 1 and Table 1). CF/PRP was significantly increased by hypoxia+acidosis but to a lesser extent compared to hypoxia alone (from 1.7 ± 0.18 μl/mmHg to 4.5 ± 0.44 μl/mmHg). Again, 5 min after start of reperfusion CF/PRP returned to baseline levels (Figure 2).

Administration of cariporide during hypoxia resulted in a less severe reduction of LV contraction parameters and to a recovery to baseline levels within 5 min. In contrast, cariporide had no influence on the hypoxia+acidosis dependent reduction in LVP, PRP and dp/dtmax (Figure 1 and Table 1).

Diastolic left ventricular function i.e., EDP and dp/dtmin also deteriorated during hypoxia and hypoxia+acidosis (Table 1). However, although the reduction of dp/dtmin was in the same magnitude in both groups, EDP increase was less severe in the hypoxia+acidosis group (18.6 ± 4.06 mmHg vs. 36.8 ± 5.44 mmHg) (Table 1). Again, after start of reperfusion, hearts treated with hypoxia+acidosis achieved their diastolic function approximately after 5 min, whereas hearts treated with hypoxia alone did not reach baseline levels after re-oxygenation.

Diastolic cardiac function was ameliorated by cariporide in the hypoxia group, in such a way that on one hand both parameters declined to a lesser extent and on the other hand recovered to control levels within 5 min of reperfusion (Table 1).

The hypoxia+acidosis group did not benefit from cariporide.

Acidosis alone did not influence EDP but significantly impaired dp/dtmin. This impairment was not affected by cariporide treatment (Table 1).

Cariporide administration during normal O_2_ concentration and normal pH did not influence cardiac hemodynamics (Figures 1, 2 and Table 1).

## Electrophysiology

**Figure 3:**
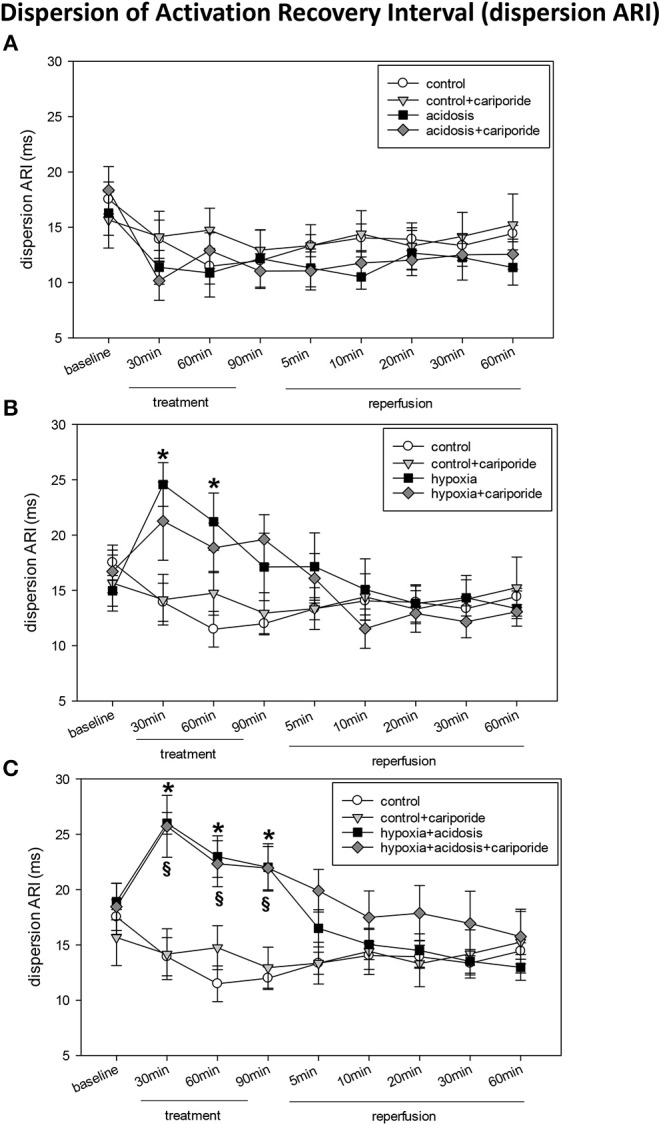
**(A)** Dispersion of activation-recovery interval (ARI) in control hearts without or with cariporide 1 μmol/L and in hearts subjected to acidosis (pH = 7.0, O_2_ = 100%) without or with cariporide 1 μmol/L. **(B)** Dispersion of activation-recovery interval (ARI) in control hearts without or with cariporide 1 μmol/L and in hearts subjected to hypoxia (pH = 7.4, O_2_ = 40%) without or with cariporide 1 μmol/L. **(C)** Dispersion of activation-recovery interval (ARI) in control hearts without or with cariporide 1 μmol/L and in hearts subjected to hypoxia+acidosis (pH = 7.0, O_2_ = 40%) without or with cariporide 1 μmol/L. All data are given as means ± SEM. Significant differences (*p* < 0.05) vs. control are indicated by a * and significant differences vs. control+cariporide by a ^**§**^.

Acidosis did not alter TAT, ARI dispersion or PTP (Figure 3 and Table 2). However, heart rate corrected ARI was significantly prolonged during the first 30 min of acidosis (from 163 ± 6.09 to 196 ± 5.52), lowered then during 60 and 90 min of acidosis to control levels and dropped significantly below control during reperfusion. The prolongation of ARI during acidosis was significantly mitigated by cariporide (Table 2).

**Table 2:** Electrophysiological data and ATP-measurements.

	**Baseline**	**Treatment**			**Reperfusion**				
		**30 min**	**60 min**	**90 min**	**5 min**	**10 min**	**20 min**	**30 min**	**60 min**
**Total activation time (TAT) in ms**									
Control	14.6 ± 0.77	13.9 ± 1.21	14.2 ± 1.21	13.9 ± 1.16	15.3 ± 0.82	15.6 ± 0.88	15.0 ± 0.95	14.8 ± 0.68	15.3 ± 0.89
Control + cariporide	15.5 ± 2.24	13.9 ± 0.92	14.3 ± 0.89	14.0 ± 0.88	14.6 ± 1.12	15.5 ± 1.59	14.3 ± 0.82	15.1 ± 0.98	14.5 ± 0.96
Acidosis	14.7 ± 1.48	13.3 ± 1.51	12.6 ± 1.38	13.5 ± 1.80	14.1 ± 1.50	13.7 ± 1.44	14.4 ± 1.97	13.3 ± 1.27	13.2 ± 1.28
Acidosis + cariporide	14.0 ± 2.25	14.8 ± 2.44	14.5 ± 2.82	15.8 ± 2.21	15.8 ± 2.36	15.7 ± 2.26	15.7 ± 2.47	15.2 ± 2.12	15.0 ± 1.92
Hypoxia	14.5 ± 2.13	17.3 ± 1.61^*^	17.0 ± 1.1^*^^#^	20.5 ± 2.67^*^^#^	20.2 ± 1.89^*^	19.8 ± 1.62^*^^#^	19.6 ± 1.57^*^^#^	18.0 ± 2.00	17.6 ± 2.20
Hypoxia + cariporide	13.9 ± 0.88	15.3 ± 1.09	14.1 ± 0.85	15.9 ± 1.11	16.7 ± 1.82	16.1 ± 0.94	15.7 ± 0.78	15.6 ± 0.78	15.0 ± 1.02
Hypoxia + acidosis	14.5 ± 1.4	17.6 ± 1.57	18.8 ± 1.06^*^	18.5 ± 1.32^*^	16.4 ± 1.01	17.7 ± 2.25	17.4 ± 1.63	15.8 ± 1.19	17.3 ± 1.56
Hypoxia + acidosis + cariporide	15.3 ± 1.1	17.9 ± 3.05	19.8 ± 3.77	18.3 ± 2.82	16.8 ± 2.00	17.2 ± 2.41	17.0 ± 2.67	16.1 ± 1.88	17.0 ± 2.76
**Activation recovery interval (ARI) in ms**									
Control	162 ± 6.23	168 ± 4.35	175 ± 5.20	178 ± 4.79	183 ± 4.11	185 ± 3.61	182 ± 4.18	181 ± 5.02	176 ± 4.96
Control + cariporide	160 ± 2.39	157 ± 2.95	157 ± 3.60	166 ± 3.63	168 ± 4.54	169 ± 4.05	172 ± 4.87	166 ± 3.70	168 ± 3.83
Acidosis	163 ± 6.09	196 ± 5.52^*^	190 ± 3.13	187 ± 3.42	152 ± 2.74^*^	159 ± 3.97	160 ± 2.37	166 ± 2.71	169 ± 3.09
Acidosis + cariporide	154 ± 3.24	180 ± 4.65^§^	183 ± 5.16	187 ± 4.72	147 ± 4.13^§^	158 ± 3.00	166 ± 5.45	170 ± 3.57	166 ± 3.25
Hypoxia	169 ± 3.04	123 ± 3.74^*^	131 ± 4.60^*^	145 ± 4.48^*^	163 ± 3.97	172 ± 3.66	180 ± 3.48	186 ± 3.55	182 ± 3.69
Hypoxia + cariporide	172 ± 3.23	123 ± 4.70^§^	126 ± 4.79^§^	131 ± 5.61^§^	158 ± 4.11	172 ± 4.23	178 ± 3.31	178 ± 3.18	183 ± 3.14
Hypoxia + acidosis	167 ± 3.89	178 ± 4.31	188 ± 3.82	184 ± 4.26	182 ± 3.05	186 ± 2.92	186 ± 2.96	189 ± 2.62	185 ± 3.21
Hypoxia + acidosis + cariporide	161 ± 5.20	168 ± 6.28	174 ± 5.49	177 ± 5.29	171 ± 4.48	172 ± 4.18	175 ± 4.09	178 ± 4.33	173 ± 3.93
**Peak to peak amplitude (PTP) in mV**									
Control	3.9 ± 0.19	3.6 ± 0.12	3.4 ± 0.12	3.3 ± 0.20	3.3 ± 0.22	3.3 ± 0.21	3.2 ± 0.21	3.2 ± 0.20	3.1 ± 0.19
Control + cariporide	3.7 ± 0.37	3.6 ± 0.41	3.5 ± 0.41	3.4 ± 0.40	3.3 ± 0.39	3.3 ± 0.39	3.2 ± 0.38	3.2 ± 0.39	3.1 ± 0.39
Acidosis	3.6 ± 0.35	3.3 ± 0.29	3.2 ± 0.27	3.1 ± 0.26	3.3 ± 0.28	3.3 ± 0.28	3.2 ± 0.27	3.3 ± 0.27	3.2 ± 0.25
Acidosis + cariporide	3.5 ± 0.25	3.2 ± 0.20	3.1 ± 0.19	3.0 ± 0.17	3.2 ± 0.17	3.2 ± 0.21	3.3 ± 0.24	3.2 ± 0.30	3.1 ± 0.20
Hypoxia	3.5 ± 0.35	2.8 ± 0.16^*^	2.6 ± 0.13^*^	2.5 ± 0.13^*^	2.7 ± 0.14^*^	2.7 ± 0.16	2.8 ± 0.16	2.8 ± 0.16	2.8 ± 0.15
Hypoxia + cariporide	3.6 ± 0.29	2.9 ± 0.17	2.7 ± 0.12^§^	2.5 ± 0.15^§^	2.6 ± 0.61	2.6 ± 0.14	2.7 ± 0.14	2.7 ± 0.13	2.8 ± 0.13
Hypoxia + acidosis	3.9 ± 0.07	2.6 ± 0.06^*^	2.5 ± 0.07^*^	2.5 ± 0.13^*^	2.6 ± 0.09	2.6 ± 0.11	2.6 ± 0.09	2.6 ± 0.1	2.7 ± 0.09
Hypoxia + acidosis + cariporide	3.9 ± 0.10	2.5 ± 0.13^§^	2.4 ± 0.14^§^	2.3 ± 0.15^§^	2.5 ± 0.15	2.5 ± 0.16	2.5 ± 0.16	2.5 ± 0.16	2.6 ± 0.16
**ATP-measurements** **μg/g tissue**									
Control									25.7 ± 6.5
Control + cariporide									15.7 ± 4.9
Acidosis									32.2 ± 9.7
Acidosis + cariporide									21.0 ± 7.5
Hypoxia									7.6 ± 2.5^*^^+^
Hypoxia + cariporide									21.8 ± 9.4
hypoxia + acidosis									31.5 ± 9.8
Hypoxia + acidosis + cariporide									30.8 ± 5.5

*All data are given as means ± SEM*.

* *significant differences vs. control*.

# *significant differences vs. hypoxia+cariporide*.

§ *significant differences vs. control+cariporide*.

+ *significant differences vs. hypoxia+acidosis*.

Hypoxia significantly prolonged TAT, enhanced ARI dispersion and reduced ARI and PTP (Figure 3 and Table 2). After about 20 min of reperfusion TAT and PTP approximated control values whereas ARI and ARI dispersion recovered after 5–10 min. Administration of cariporide during hypoxia significantly reduced TAT prolongation, but did not alter ARI, ARI dispersion or PTP reduction (Figure 3 and Table 2).

In case of hypoxia+acidosis TAT and ARI dispersion were significantly increased and PTP decreased (Figure 3 and Table 2) [from 14.5 ± 1.4 to 18.5 ± 1.32 (TAT); from 18.9 ± 1.65 to 22.0 ± 2.14 (ARI dispersion) and from 3.9 ± 0.07 to 2.5 ± 0.13 (PTP)]. ARI itself was slightly increased by hypoxia+acidosis, however without reaching significance. Reperfusion with normal Tyrode's solution led to a normalization of TAT, ARI dispersion and PTP within 5 min. Cariporide administration had no influence on the increase or decrease of electrophysiological parameters.

Interestingly, under control conditions cariporide itself led to a slight reduction of ARI and heart rate, however, this reduction was not significant (Figures S1, S2).

The propagation of the activation wave front also exhibited changes with hypoxia and acidosis. This became obvious from changes in the self-similarity of vector fields during treatment as compared to those under control conditions. The maximum beat-to-beat similarity of length and direction of the vectors was nearly 60% under control conditions. Subsequent beats changed their pattern so that the similarity to baseline condition dropped to about 35% (control group, 60 min), to 24% (60 min of hypoxia) or to 34% (60 min of acidosis), or to 30% (60 min of hypoxia+acidosis). During reperfusion vector field similarity did not return to control levels in the hypoxia group. In contrast, in the hypoxia+acidosis group control levels were reached after 5 min of reperfusion.

**Figure 4:**
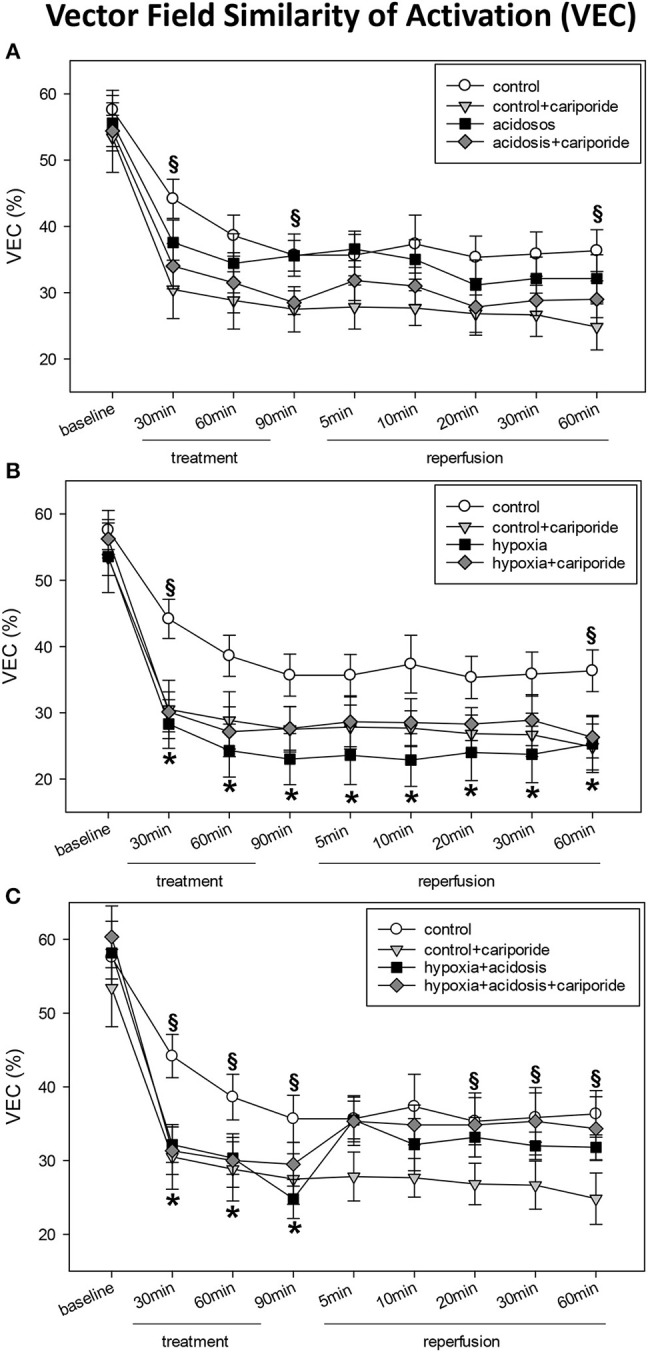
**(A)** Vector field similarity of activation (VEC) in control hearts without or with cariporide 1 μmol/L and in hearts subjected to acidosis (pH = 7.0, O_2_ = 100%) without or with cariporide 1 μmol/L. **(B)** Vector field similarity of activation (VEC) in control hearts without or with cariporide 1 μmol/L and in hearts subjected to hypoxia (pH = 7.4, O_2_ = 40%) without or with cariporide 1 μmol/L. **(C)** Vector field similarity of activation (VEC) in control hearts without or with cariporide 1 μmol/L and in hearts subjected to hypoxia+acidosis (pH = 7.0, O_2_ = 40%) without or with cariporide 1 μmol/L. All data are given as means ± SEM. Significant differences (*p* < 0.05) vs. control are indicated by a *, and significant differences vs. control+cariporide by a **§**.

Cariporide had only minor effects on changes in vector field similarity (Figure 4).

### Histological Analysis

Hypoxia/reperfusion is known to be able to induce myocardial dysfunction by generation of free radicals and induction of apoptosis. Thus, we wanted to clarify whether these processes might be involved in the effects of cariporide on hypoxia and/or acidosis.

**Figure 5:**
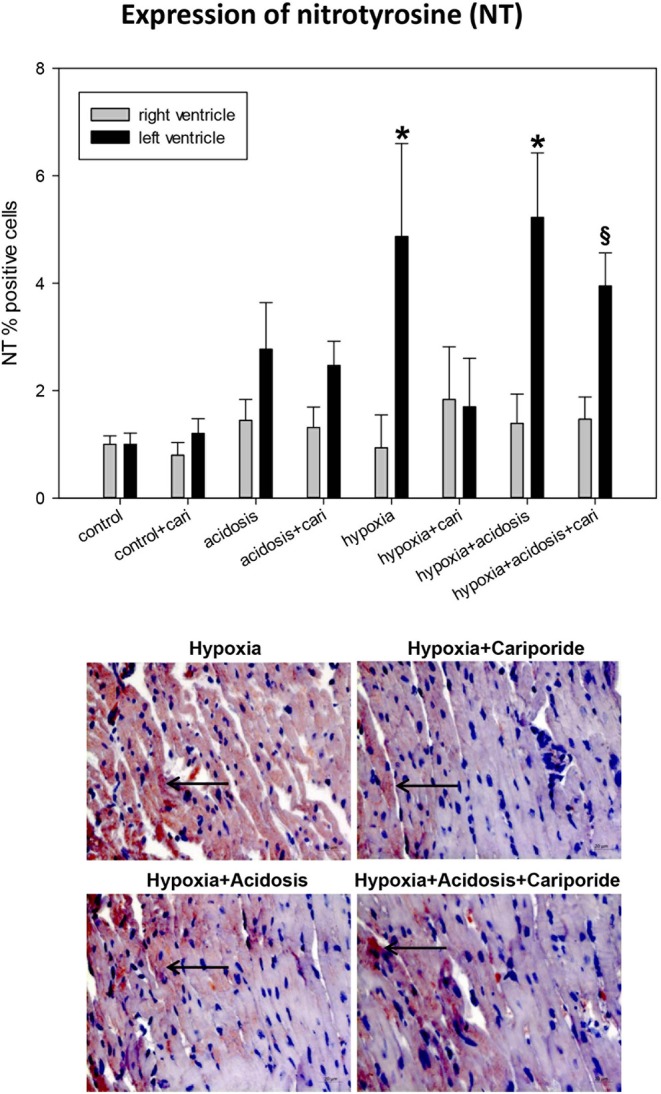
**(Upper)** NT (nitrotyrosine) expression in ventricular tissue. All data are given as means ± SEM. Significant differences (*p* < 0.05) vs. control are indicated by a *, and significant differences vs. control+cariporide by a ^**§**^. cari, cariporide. **(Lower)** Original pictures of NT immunohistology (LV). Arrows point toward positive cells.

Peroxynitrite radicals induce nitrosylation of tyrosine residues, which can be detected as nitrotyrosines. During hypoxia and hypoxia+acidosis nitrotyrosine formation was significantly elevated in left ventricle. This elevation was blocked by cariporide application during hypoxia. In contrast, cariporide had no influence on the hypoxia+acidosis associated NT elevation (Figure 5).

**Figure 6:**
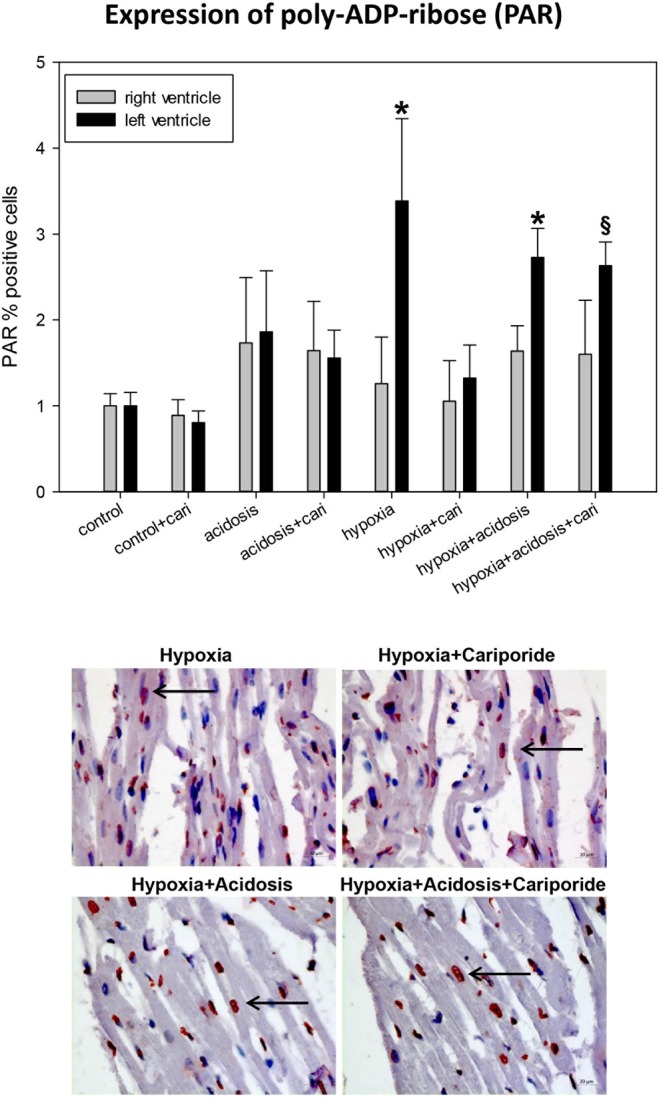
**(Upper)** PAR (poly ADP-ribose) expression in ventricular tissue. All data are given as means ± SEM. Significant differences (*p* < 0.05) vs. control are indicated by a *, and significant differences vs. control+cariporide by a ^**§**^. cari, cariporide. **(Lower)** Original pictures of PAR immunohistology (LV). Arrows point toward positive cells.

Free radicals are also able to produce DNA strand breaks leading to the activation of an energy consuming repair mechanism by poly-ADP-ribose polymerase (PARP). We have investigated the end-product of this process, PAR. PAR nuclear translocation significantly increased during hypoxia and hypoxia+acidosis in left ventricular specimen. Again cariporide lowered PAR in case of hypoxia but not in case of hypoxia+acidosis (Figure 6).

**Figure 7:**
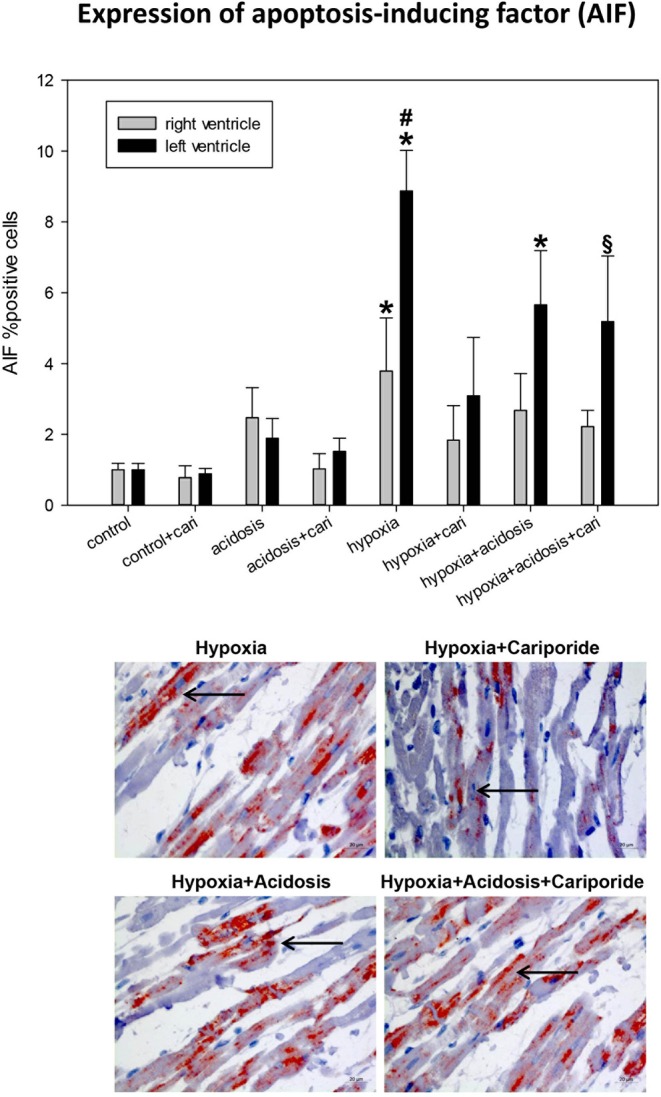
(Upper) AIF (apoptosis inducing factor) expression in ventricular tissue. All data are given as means ± SEM. Significant differences (*p* < 0.05) vs. control are indicated by a *significant differences vs. control+cariporide by a ^**§**^ and significant differences vs. hypoxia+cariporide by a ^**#**^. cari, cariporide. **(Lower)** Original pictures of AIF immunohistology (LV). Arrows point toward positive cells.

Accumulation of PAR can -if parallel to ATP decrease- lead to the translocation of AIF to the nucleus. Histological analysis revealed that hypoxia and hypoxia+acidosis led to a significant AIF nuclear translocation in left and to a lesser extent also in right ventricular specimen. Acidosis alone or cariporide application during control conditions did not significantly alter AIF translocation (Figure 7). Cariporide treatment during hypoxia reduced AIF elevation, whereas in case of hypoxia+acidosis cariporide did not decrease AIF.

**Figure 8:**
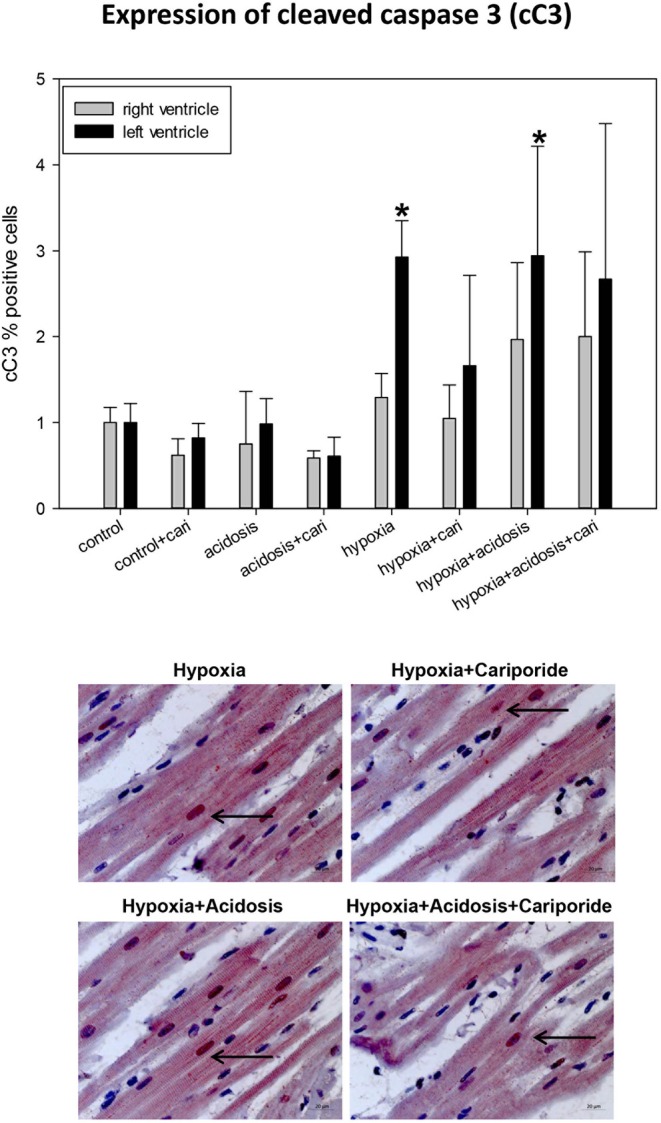
**(Upper)** cC3 (cleaved caspase 3) expression in ventricular tissue. All data are given as means ± SEM. Significant differences (*p* < 0.05) vs. control are indicated by a *. cari, cariporide. **(Lower)** Original pictures of cC3 immunohistology (LV). Arrows point toward positive cells.

If the extrinsic apoptosis pathway is initiated an early step in the cascade is the activation of caspase 3 by cleavage, as can be detected by cC3 immonohistology. Significant cC3 nuclear translocation was found in left ventricular specimen during hypoxia and hypoxia+acidosis. But generally, the number of cC3 positive cells was small and did not exceed 5% (Figure 8).

### ATP Analysis

At the end of the experiments, i.e., after reperfusion, samples of the left ventricle were shock frozen and analyzed for the tissue ATP content (see methods). As shown in Table 2, we found that despite the 60 min reperfusion period in hypoxia-treated hearts ATP was still decreased. Additional cariporide resulted in unchanged ATP as compared to control. Acidosis alone and in combination with hypoxia lead to an increase in tissue ATP. Cariporide did not exhibit effects on ATP under the combination of hypoxia and acidosis.

## Discussion

The data of the present study show differential effects of hypoxia and acidosis on cardiac hemodynamics and electrophysiology.

It is clinically well-known that cardiac ischemia results in reduced contractile force. Accordingly, we found a reduction in LVP and dp/dt max in hypoxia and to a lesser extent in acidosis. Interestingly the combination of acidosis and hypoxia resulted in less severe loss in contractility in particular during recovery indicating a possible mitigating effect of acidosis on hypoxia, which should be discussed here.

Hypoxia leads to less ATP-generation as can be seen from the final ATP tissue level (see Table 2). Acidosis counteracted this effect, which can be explained by the finding that acidosis prevents from the formation of mitochondrial transition pores and from mitochondrial hypoxia-induced injury (Baines, 2009; Chi et al., 2019).

Ischemia-reperfusion injury typically leads to formation of nitrotyrosine, activation of PARP with consecutive PAR production, nuclear translocation of AIF and cleavage of C3, which are processes involved in the early initiation of apoptosis. These effects are known to be mainly induced by free radicals. Since the free oxygen-derived radicals are formed during reperfusion following hypoxia, and since the additional acidosis-treatment (hypoxia+acidosis) was not continued during the reperfusion period, an effect of acidosis on radical-induced processes like PAR-formation and protein-nitrosylation is unlikely and accordingly has not been observed in our study.

AIF and C3 cleavage are consequences of previous PAR formation and free radicals (Wang et al., 2009, 2014), so that these alterations also are not expected to be affected by the additional acidosis treatment for the same reasons as mentioned above.

Regarding the electrophysiological effects of acidosis, hypoxia and their combination, our data is in good accordance with cellular investigations using voltage clamp techniques. Kanaporis et al. (2017) found that metabolic inhibition reduces I(Ca.L) (L-type calcium current). The authors attributed the reduction in I(Ca.L) to the occurrence of intracellular acidosis, due to the reverse mode of mitochondrial ATP-synthase during metabolic inhibition. Elevation of intracellular pH alleviated this effect. Thus, inhibition of I(Ca.L) probably contributes to the loss in contractility in ischemia. The effect of metabolic inhibition on I(Ca.L) however is biphasic (Treinys et al., 2019): in the initial phase of metabolic inhibition I(Ca.L) is increased followed by the known decrease in I(Ca.L) during continued metabolic inhibition. These observations of a reduction in I(Ca.L) by acidosis and metabolic inhibition can help to explain the loss of contractile force seen in our experiments.

However, the combined effect of acidosis and hypoxia requires additional considerations. In our study additional acidosis lead to a faster recovery of most hypoxic-induced changes in hemodynamics and electrophysiology but did not (or only marginally) affect hypoxia-induced pro-apoptotic changes as AIF-translocation, C3 cleavage, NT-formation or PAR-synthesis.

Regarding the electrophysiological changes, on the one hand it was shown that extracellular acidosis can reduce I(Ca.L) (Cheng et al., 2009). On the other hand Cheng et al. (2009) showed that extracellular acidification [pH(extracellular): from 7.4 to 6.8 or 6.3] slowed spontaneous action potential rate and upstroke velocity, and reduced maximum diastolic potential as well as action potential amplitude (Cheng et al., 2009). This was due to pH(extracellular) dependent decrease in L-type Ca current [I(Ca.L)]. Moreover, acidosis also affects the shape of the action potential. This involves also effects on I(K.r) (rapid delayed rectifier potassium current) and I(t.o) (transient outward potassium current) (Cheng et al., 2009). Extracellular acidosis reduced the amplitude of I(K.r), so that in a subsequent study the authors concluded that the percentage of contribution to action potential repolarization by I(K.r) is reduced and that I(K.r) may then have a lower counteracting impact on pro-arrhythmogenic depolarizing stimuli (Du et al., 2010).

Interestingly, Saegusa et al. (2013) observed differential effects of intracellular and extracellular acidification on the action potential duration: intracellular pH reduction resulted in action potential prolongation while extracellular acidosis reduced action potential duration (Saegusa et al., 2013). These authors found that intracellular acidification reduced the transient outward current I(t.o.) and I(Ca.L) during the action potential. Extracellular acidification shifted I(t.o.) inactivation toward less negative potentials (Saegusa et al., 2013).

Another important effect is that regarding the ATP-inhibited K^+^ current I(K.ATP) (ATP-sensitive potassium current) it was described that hypoxia and ATP depletion can activate this current leading to action potential shortening (Wilde et al., 1990) and that -on the other hand- extracellular H^+^ ions can inhibit I(K.ATP) (Moncada et al., 2000). This is in good accordance to our present observation that ARI was shortened by hypoxia but not by the combination hypoxia+acidosis. This may -at least partially- be explained by the antagonization of hypoxia-induced ATP-loss (see Table 2; from 25.7 to 7.6 μg/g) by additional acidosis (see Table 2: hypoxia+ acidosis 31.5 μg/g).

Taken together, with regard to the data of our study, these studies could mean that hypoxia leads to reduced Ca^2+^ influx with shortened action potential and that additional acidosis may alleviate this effect by prolonging action potential duration [via reduction of I(K.ATP), I(t.o.) and I(K.r)], which prolongs the time for I(Ca.L) influx.

In accordance with these considerations the effects of acidosis on EDP are less pronounced than those of hypoxia and the combined effect is mitigated in comparison to hypoxia alone.

Regarding the propagation of the cardiac action potential, this is dependent on I(Na) (fast sodium current) availability and on gap junction coupling. The parameter related to ventricular conduction is total activation time (Dhein et al., 1993). The present data show an increase in TAT, i.e., slowing of conduction, in hypoxia, which can be explained by a reduced I(Na) availability in hypoxia which may result from depolarization due to ATP-depletion induced reduction in Na^+^/K^+^-ATPase activity (Buchanan et al., 1985; Janse, 1986; Kléber et al., 1986). A change in TAT can be expected to result in a change of the activation pattern, i.e., of the propagation vectors. Accordingly, this could be verified in the present study by reduced vectorfield similarity during hypoxia, showing changes in the direction and velocity of the activation vectors.

I(Na) also can be reduced by H^+^ ions (Murphy et al., 2011). By comparison of the effects of intracellular and extracellular acidification Watson and Gold (1995) found that reduction of peak I(Na) is a function of pH(extracellular), while steady-state inactivation was modulated by pH(intracellular) (Watson and Gold, 1995). In these experiments the time course of activation and inactivation depended on both pH(extracellular) and pH(intracellular). Extracellular acidification led to a 31% decrease in peak I(Na). However, the acidified pH(extracellular) in these experiments was about 6.5, which is much lower than in our experiments. Accordingly, we did not observe a clear depressant effect of acidosis on TAT. However, a small effect on vectorfield similarity was observed showing that there was a slight deviation of the activation vectors. It should be noted at that point that activation vectors depend not only on I(Na) and gap junction opening but also on the foregoing repolarization since this affects I(Na) availability.

The other factor regulating ventricular conduction is the opening of gap junctions. These also close at very low pH near 6.5 (Hagen et al., 2009), and moreover, estimations calculate that nearly 50% of the gap junctions must be closed to achieve a slowing of conduction (van Rijen et al., 2006). Thus, it is reasonable that in the present experiments acidosis alone did not slow conduction. This is also in line with the only small effect of acidosis as used in our experiments on VEC. In accordance with the only small effects on VEC, we did not observe sustained ventricular arrhythmia [according to previous studies arrhythmia occurs at VEC values below 10%; Dhein et al., 1990, 1993]. For experiments aiming at arrhythmia-induction additional pro-arrhythmic factors need to be introduced such as reduced K^+^, or stronger acidification.

Another aspect is the heart rate. This parameter was only slightly affected in our study. This is in line with the observation that I(f) (funny current) is unaffected by acidosis (Cheng et al., 2009).

The next aspect to be considered is the effect of cariporide. Cariporide, formerly known as Hoe642, inhibits the cardiac NHE. This inhibition requires an activation of this pump. NHE typically transports H^+^ out of the cell and increases Na^+^ uptake. Thus, intracellular acidification activates this pump (Lazdunski et al., 1985). In consequence, the effects seen with cariporide in acidosis in our experiments (which is an extracellular acidosis) were very small and almost not significant. Regarding the activation pattern, we found that the effect of hypoxia was slightly mitigated by simultaneous extracellular acidosis, which may be explained by the idea that high extracellular H^+^ concentration reduces the driving force for the NHE, thus reducing the intracellular Na^+^ overload.

If however, the intracellular pH drops then NHE should be activated. This can be expected in hypoxia, resulting in Na^+^ overload and secondary Ca^2+^-overload by activation of the Na^+^/Ca^2+^exchanger. This Ca^2+^-overload, which typically leads to cardiac dysfunction, has been shown to be antagonized by NHE1-inhibition (Anzawa et al., 2012; Uthman et al., 2018b; for recent review see: Uthman et al., 2018a)

If so, one would expect that inhibition of NHE by cariporide should mitigate the effects of hypoxia on cardiac electrophysiology and hemodynamics. This was indeed seen in the data.

Interestingly, the hypoxia-induced changes in AIF, cC3, NT, and PAR were also reduced by cariporide. Since NHE1 inhibition leads to lower Ca^2+^-overload by Na^+^/Ca^2+^-exchanger, which is an energy consuming process, NHE1-inhibition may result in an ATP-sparing effect (Anzawa et al., 2012; Counillon et al., 2016; Uthman et al., 2018a). In support of this hypothesis we found indeed higher ATP levels if hypoxia-treated hearts received cariporide (see Table 2). Other mechanisms yet unknown may also contribute (Counillon et al., 2016).

If hypoxia however was combined with (extracellular) acidosis, the effects of cariporide were diminished. This can be explained by the assumption that due to the enhanced extracellular H^+^ concentration there was no H^+^ gradient so that NHE probably was not, or less activated, and cariporide could not exhibit effects, or only small effects (Bonde and Boedtkjer, 2017).

This is however part of the clinical situation in which in cardiopulmonary arrest a general hypoxia is combined with increasing lactate levels and often reduced breathing, which means an extracellular acidosis. Thus, the situation simulated in our study may at least in parts resemble this clinical setting and can help to explain why cariporide in clinical studies was less effective than previously foreseen.

## Conclusions

This study shows differential effects of hypoxia and acidosis on cardiac performance and electrophysiology. Moreover, it demonstrates that extracellular acidification can mitigate the effects of hypoxia. Regarding the NHE inhibitor cariporide, the data indicate that this drug acts mainly on the effects caused by an isolated hypoxia but shows less effect in simultaneous presence of extracellular acidosis. This, however, may help to explain why cariporide in clinical studies was less effective than expected, since in the situation of acute myocardial infarction and cardiogenic shock extracellular acidosis is a contributing factor.

## Data Availability Statement

All datasets generated for this study are included in the article/Supplementary Material.

## Ethics Statement

The animal study was reviewed and approved by Landesdirektion Sachsen, Braustraße 2, 04107 Leipzig.

## Author Contributions

AS conceived and designed the experiments, analyzed the data, and wrote the manuscript. MJ, HZ, BS, and JV performed the experiments and analyzed the data. ID, GA, and JS conceived the experiments. SD designed the experiments, analyzed the data, and edited the manuscript.

### Conflict of Interest

The authors declare that the research was conducted in the absence of any commercial or financial relationships that could be construed as a potential conflict of interest.

## Abbreviations

AIF: Apoptosis inducing factor
ARI: Activation recovery interval
ATP: Adenosine triphosphate
BCL: Basic cycle length
cC3: Cleaved caspase 3
CF: Coronary flow
dp/dt(max): Maximum contraction velocity
dp/dt(min): Maximum relaxation velocity
ECG: Electrocardiogram
HR: Heart rate
I(Ca. L): L-type calcium current
I(f): Funny current
I(K.ATP): ATP-sensitive potassium current
I(K.r): Rapid delayed rectifier potassium current
I(Na): Fast sodium current
I(t.o): Transient outward potassium current
LV: Left ventricle
LVP: Left ventricular pressure
NHE1: Sodium/proton exchanger 1
NT: Nitrotyrosine
PAR: Poly-ADP ribose
PARP: Poly-ADP ribose polymerase
PTP: Peak-to-peak amplitude
pO_2_: Partial oxygen pressure
PQ: PQ-interval
PRP: Pressure rate product
RV: Right ventricle
TAT: Total activation time
VEC: Vector field similarity of activation.
