# Supplementary material for: Effects of Hypoxia and Acidosis on Cardiac Electrophysiology and Hemodynamics. Is NHE-Inhibition by Cariporide Still Advantageous?
Source: Front Physiol. 2020 Mar 19;11:224. doi: 10.3389/fphys.2020.00224 (PMC7103633; doi:10.3389/fphys.2020.00224)
Supplement: Supplementary file 1 [file Data_Sheet_1.docx]

**Supplement**

Supplement figure S1

Figure S1: BCL (basic cycle length) in ms without or with cariporide 1µmol/L. Cariporide slightly but not significantly prolonged BCL (i.e. lowered heart rate).

Supplement figure S2

Figure S2: ARI (activation recovery interval, frequency corrected) in ms without or with cariporide 1µmol/L. Cariporide slightly but not significantly lowered ARI.
